# Predicting non-small cell lung cancer-related genes by a new network-based machine learning method

**DOI:** 10.3389/fonc.2022.981154

**Published:** 2022-09-20

**Authors:** Yong Cai, Qiongya Wu, Yun Chen, Yu Liu, Jiying Wang

**Affiliations:** ^1^ Department of Radiation Oncology, Shanghai Pulmonary Hospital, Tongji University School of Medicine, Shanghai, China; ^2^ Department of Oncology, Shanghai Pulmonary Hospital, Tongji University School of Medicine, Shanghai, China

**Keywords:** lung cancer, computational techniques, deep walk, graph convolutional network, deep neural network

## Abstract

Lung cancer is the leading cause of cancer death globally, killing 1.8 million people yearly. Over 85% of lung cancer cases are non-small cell lung cancer (NSCLC). Lung cancer running in families has shown that some genes are linked to lung cancer. Genes associated with NSCLC have been found by next-generation sequencing (NGS) and genome-wide association studies (GWAS). Many papers, however, neglected the complex information about interactions between gene pairs. Along with its high cost, GWAS analysis has an obvious drawback of false-positive results. Based on the above problem, computational techniques are used to offer researchers alternative and complementary low-cost disease–gene association findings. To help find NSCLC-related genes, we proposed a new network-based machine learning method, named deepRW, to predict genes linked to NSCLC. We first constructed a gene interaction network consisting of genes that are related and irrelevant to NSCLC disease and used deep walk and graph convolutional network (GCN) method to learn gene–disease interactions. Finally, deep neural network (DNN) was utilized as the prediction module to decide which genes are related to NSCLC. To evaluate the performance of deepRW, we ran tests with 10-fold cross-validation. The experimental results showed that our method greatly exceeded the existing methods. In addition, the effectiveness of each module in deepRW was demonstrated in comparative experiments.

## 1 Introduction

Lung cancer continues to be the primary cause of cancer deaths worldwide, causing 1.8 million fatalities annually (1). The two primary kinds of lung cancer are small cell lung cancer (SCLC) and non-small cell lung cancer (NSCLC). Additionally, nearly 85% of all cases of lung cancer are related to NSCLC (2). More and more researchers found that lung cancer is highly inherited and is associated with certain genes that increase the risk (3).

Genome-wide association studies (GWAS) are a common method to mine diseased-related genes. Hung et al. (4) firstly used GWAS and found a locus in chromosome region 15q25 that related to lung cancer. Hu et al. (5) reported that 5p15 locus is related to lung cancer *via* GWAS, and 6p21 was found by Wang et al. (6). With the development of next-generation sequencing (NGS), whole-exome sequencing (WES), whole-genome sequencing (WGS), and other technologies are applied to find disease-related genes. Sun et al. (7) applied WES on 73 advanced NSCLC tumor samples and demonstrated Protein tyrosine phosphatase receptor type D (PTPRD) might be both a prognostic and a predictive biomarker predicting clinical outcomes in non-squamous (ns)-NSCLC patients. Liu et al. (8) found infrequent detrimental mutations in GWAS-nominated sites in dopamine β-hydroxylase (DBH) and coiled-coil domain containing 147 (CDC147) *via* WES.

With the explosive growth of relevant information and data in recent years, GWAS and other methods become more and more time-consuming and laborious. Many studies have focused on drug–disease association tasks and other bioinformatics tasks through machine learning and deep learning methods (9–13). Graph neural network methods that can integrate multiple types of knowledge bases are suitable for this task. 14) used graph convolutional network (GCN) to capture structural information from the network integrating gene and disease. GCN (15) is one type of neural network architecture to learn nodes and edges of graphs. It has been proven that GCN enhances algorithms of abilities to mine information and make decisions in the bioinformatics field like Deep-DRM (16).

Graph embedding methods are popular in this task. Xiong et al. (17) built a heterogeneous network that incorporates different type datasets and obtained network representation by random walk (RW) to predict gene–disease associations. RW is a common graph embedding approach. This approach has been used to research microRNAs (miRNAs) (18), gene expression (19), and drug repositioning (20). Deep walk (21) is a graph structure data-mining algorithm that combines RW and work2vec. Zhu et al. (22) integrated graph embedding representation and GCN to learn the gene–disease associations. They connected the two methods in series as the encoder to learn features and predicted associations by a decoder.

In the paper, we focused on the problem of mining NSCLC-causing genes. We treated it as a binary classification and proposed a new network-based method. We integrated two types of graph embedding method, deep walk and GCN, to represent the gene interaction network and learn the features and used DNN to predict which genes are related to NSCLC.

## 2 Methods

**Figure 1:**
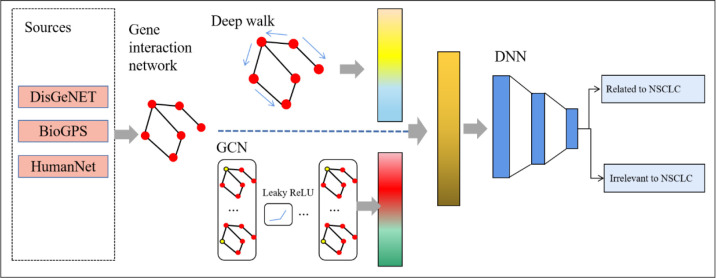
The structure of deepRW. GCN, graph convolutional network; DNN, deep neural network.

We proposed a new method named deepRW based on the gene interaction network to predict NSCLC-related genes. The structure of our method is shown in **Figure 1**. First, we built a graph network that represented the interactions between genes. Then, we utilized two types of graph embedding method, deep walk and GCN, to learn network information and extract features. Last, we constructed a DNN module to predict disease-related genes.

### 2.1 Construction of the gene network

The network of gene interactions is represented as a graph network. The graph network we built can be expressed as G= (V, E) V represents the genes that we selected related to NSCLC; E represents the interactions between genes. It should be emphasized that outliers that did not interact with other genes were eliminated.

### 2.2 Network representation by deep walk and graph convolutional network

After we obtained the gene interaction network G= (V, E), we used two graph embedding methods to learn the representations of vertices.

#### 2.2.1 Network representation by deep walk

Deep walk uses randomness to produce the sequences of vertices [*v*_1_, *v_2_,… v_n_*], where *v_i+1_* is a vertex picked at random from the neighbors of vertex *v_i_*, and the likelihood of choosing each neighbor is proportional to the weight of the edge in the adjacency matrix that corresponds to it. In the paper, we were able to build sequences at each vertex by using deep walk.

Skip-gram (23) was used to train on the sequences of the vertices by sliding window sampling. Deep walk is actually a combination of RW and skip-gram. RW is responsible for sampling to obtain the co-occurrence relationship between nodes in the graph. Skip-gram trains the embedding vectors of nodes from the relationship. After training, we can get the embedding representation vectors and the probability distribution of the vertices. A representation vector optimizes the conditional probability P*(*v_c_/v_i_ *)*, where *v_c_* is the vertex that is in the context window of *v_i_*. The loss function of training is:


(1) $$L_{vi}=-logP(v_{c1},v_{c2},\ldots ,v_{cW}│v_{i})=-log\prod_{j=1}^{W}P(v_{cj}│v_{i})$$

where W represents the window size.

#### 2.2.2 Network representation by graph convolutional network

The other graph embedding method we used is GCN. GCN used the graph network to learn node and edge information of the graph. Compared with deep walk, GCN can not only learn the structure of each node and its neighborhood but also integrate the characteristics of each node into it. If A is the adjacency matrix, the Laplacian matrix is:


(2) L=D−A

where D means the degree matrix of the network. Since the features of genes should contain not only connections between nodes but also the information itself. So we can get:


(3) $$A^{\prime }=A+I$$

where I is the identify matrix. Then, the inverse degree matrix *D* ^′^ can be obtained.


(4) $$D^{\prime }=\sum^{}A^{\prime }$$

Last, we can get the features as follows:


(5) $$X^{\prime }=\;\sigma (D^{'\frac{1}{2}}A^{\prime }D^{'\frac{-1}{2}}X)$$

where X is the feature vector of each vertex, and σ is the activation function. In the study, we used Leaky Rectified Linear Unit (Leaky ReLU) function (24) as the activation function. This activation function may reduce the likelihood of vanishing gradients and boost feature sparsity when compared to other activations. The formula is as follows:


(6) LeakyReLU(x)=max(0,x)+0.2min(0,x)

Two feature vectors of each vertex were generated *via* deep walk and GCN. Then, two feature vectors were fused and delivered to the prediction module.

### 2.3 Network prediction by deep neural network

To increase the quality of features and determine whether or not the gene is related to NSCLC, we employed a DNN module after network representation by deep walk. Whether there is a linear or non-linear connection between the input and the output, DNN can determine the appropriate mathematical operation to convert the input into the output. Now, most classification methods are shallow structure algorithms, which have the disadvantages of limited representation ability of complex functions in the case of limited samples and calculation suits, and the generalization ability for complex classification problems is limited. Deep learning can realize complex function approximation by learning a deep non-linear network structure and represent the distributed representation of input data. DNN has stronger ability to abstract problems and can also simulate more complex models. The following formula may be used to determine the feature map that advances to the next layer:


$$Output^{l}=\;W^{l}Input^{l}+Bias^{l}$$

where Input is the input of the forward propagation, Output is the output, Bias is the bias of layer l, and W is the weight of the neurons. The output of each layer is then sent *via* an activation function, which boosts positive vectors and suppresses negative vectors from the previous layer. We still used Leaky ReLU as the activation function in the predicting module.

**Figure 2:**
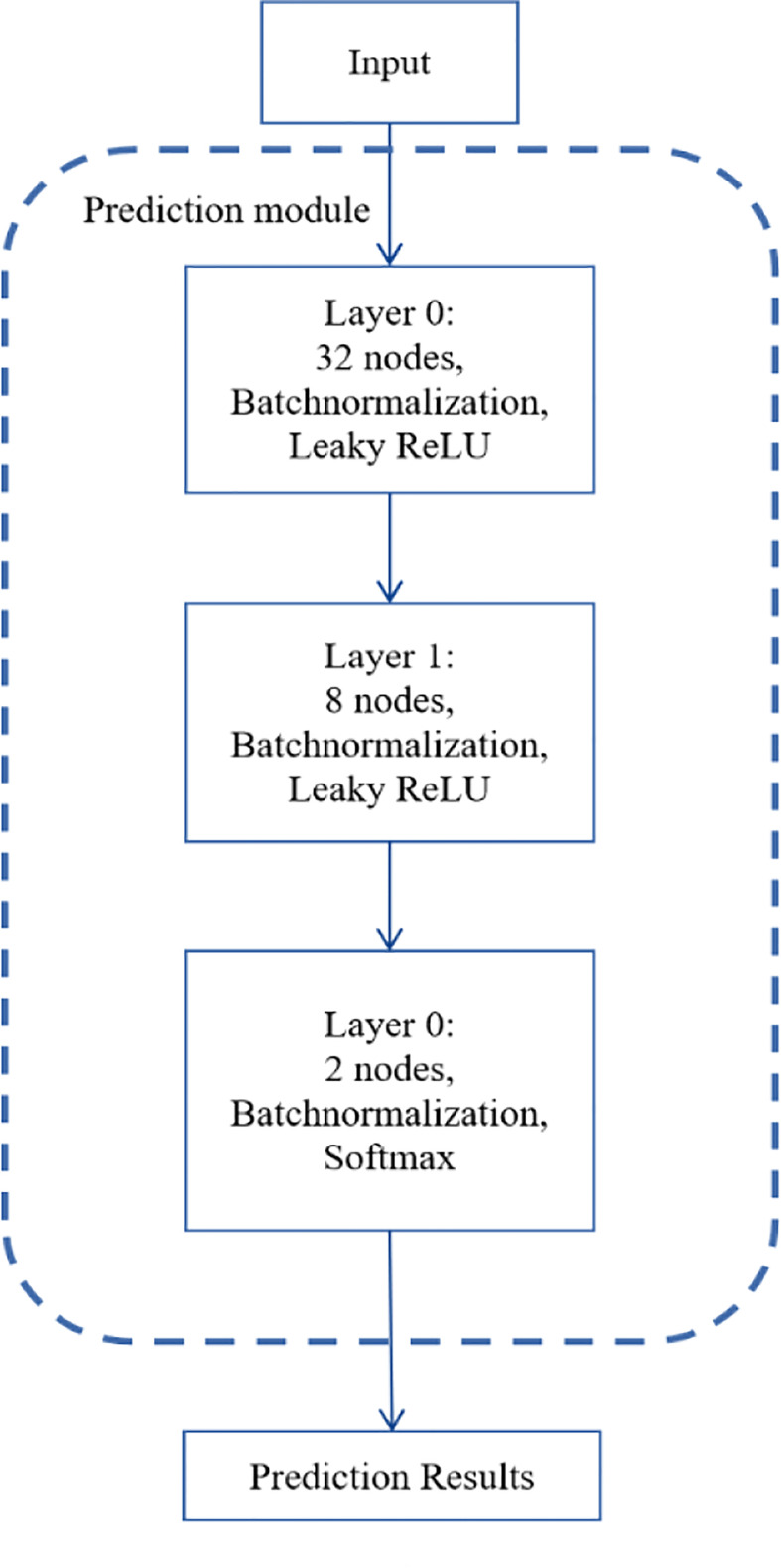
The structure of the DNN module.

**Figure 2** depicts the number of layers of the DNN module and the specific parameters of each layer. There are three layers in the DNN module. Identifying NSCLC-related genes is a binary classification task, so we applied softmax as the activation function of the output layer. We used binary cross-entropy as the loss function as follows:


(7) $$\text{Loss}=\;-y_{i}log(p_{i})-(1-y_{i})log(1-p_{i})$$

where *y_i_* means the true value, *p_i_* means the predicted value. Also, we used batch normalization (25) and early stop in the training process, which can end training if no improvement is shown after 50 epochs.

## 3 Materials

### 3.1 Dataset

DisGeNET (26) is used to obtain the gene–disease associations. DisGeNET is a database that contains information on the links between genes and disease. It is one of the biggest collections of genes and variants linked with human diseases. The data in DisGeNET come from a variety of sources, including expert-curated archives, catalogs of GWAS, animal models, and published scientific articles.

HumanNet (27), a probabilistic functional gene database, is used to generate the gene–gene associations; each gene–gene association has a score that represents the probability of the association. The gene network can be expressed as *G^gg^=* (*N^gg^, E^gg^*), where *N^gg^* is the set of genes and *E^gg^* is the association of genes. The adjacent matrix of *G^gg^* is *W^gg^* ∈ *R^N×N^*, where $W_{ij}^{gg}=W_{ji}^{gg}=w$ , w is the weight of each association of genes provided by HumanNet (**Supplementary Table 1**).

In the paper, we found 142 genes linked to NSCLC from DisGeNET, containing stage I, II, III, IIIA, and IIIB types(Supplementary table 1). These 142 genes were positive samples, and another 142 genes were randomly chosen that were reported to be irrelevant to the NSCLC disease. We used gene expression of tissues as the gene features from BioGPS (28).

### 3.2 Experimental setup

To demonstrate the performance of deepRW, we utilized 10-fold cross-validation to repeat experiments 10 times. The dataset is separated into 10 subsets, in every time experiment, we randomly choose one subset as the test samples, and the others as the train samples. The precision-recall curve (AUPR) and area under the ROC curve (AUROC) is used to evaluate the effectiveness of the methods.

In the training set, the main hyper parameters were set as follows: the window size of the Skip-gram was set to 10, and Skip-gram was trained for 10 iterations. The GCN of three layers and DNN module was trained 50 epochs, and early stopping and Adam with default parameters were used.

To demonstrate the effectiveness of our method, we tested the performance of our method by comparing the models listed as follows.

**RWR:** Random walk with restart (29) is used to capture relationships between two nodes and the overall structure information of the network by calculating the proximity between two nodes.

**KBMF:** Kernelized Bayesian matrix factorization (30), which always is used in recommender systems, can take advantage of many side information sources.

**RF:** Random forest (31) is a classifier that contains multiple decision trees, and its output is determined by the votes on individual trees.

## 4. Result

### 4.1 Performance of deep walk and graph convolutional network

First, we discussed the influence of the number of GCN layers. It is known that stacking too many layers into a GCN causes the vanishing gradient problem. This means that back-propagating through these networks leads to over-smoothing, eventually leading to features of graph vertices converging to the same value (32). We constructed the GCN module with two layers, three layers, and four layers. The results are shown in **Table 1**. From the results, GCN with three layers obtained the highest scores. Stacking four layers slightly reduced performance because of over-smoothing. In the paper, we built GCN with three layers as an encoder.

**Table 1:** The effectiveness of deep walk and GCN in deepRW.

Number of layers	AUROC	AUPR
Two layers	0.702	0.723
Three layers	0.763	0.795
Four layers	0.741	0.769

AUROC, area under the ROC curve; AUPR, The area under the precision recall curve.

Then, we demonstrated the effectiveness of each module through the comparison trial on our method missing specific module. In “Without GCN,” we only used deep walk as the network representation module. In “Without deep walk,” we only used GCN. In “DNN,” we directly used DNN as the encoder and the decoder. **Table 2** shows the results. Without GCN or deep walk, our method obtained worse scores. We can conclude that GCN and deep walk are important parts in deepRW, and integrating deep walk and GCN can improve the ability of learning the graph network.

**Table 2:** The AUROC and AUPR scores of different methods.

Method	AUROC	AUPR
DeepRW	0.763	0.795
KBMF	0.701	0.748
RF	0.647	0.697
RWR	0.636	0.659

DeepRW, Deep random walk; KBMF, Kernelized Bayesian matrix factorization; RF, Random forest; RWR, Random walk with restart.

### 4.2 Performance of different methods

To decrease the errors, we repeated the experiment 10 times and calculated the average scores as the final results. From the results in **Table 3**, we can find that deepRW outperformed all other methods in terms of AUROC and AUPR scores of 0.763 and 0.795. The RWR obtained the worst scores with AUROC and AUPR of 0.636 and 0.659, which are lower than deepRW by 16.64% and 17.11%. The results demonstrated that deepRW works better than a number of machine learning methods for locating NSCLC-related genes. GCN and deepRW are the methods that can extract feature information of nodes and edges. The results show that interactions between genes are helpful for enriching the characteristic information of genes. Compared with RWR, deep walk had better performance because deep walk combines RW and word2vec, which makes the algorithm easier to converge. Although deepRW obtained the best performance in the task, this method needs a long time to train and needs more train data to get better results.

**Table 3:** The AUROC and AUPR scores of different methods.

Method	AUROC	AUPR
DeepRW	0.763	0.795
KBMF	0.701	0.748
RF	0.647	0.697
RWR	0.636	0.659

DeepRW, Deep random walk; KBMF, Kernelized Bayesian matrix factorization; RF, Random forest; RWR, Random walk with restart.

## 5 Conclusion

Lung cancer is the leading cause of cancer death globally, and NSCLC is the main pathological subtype of lung cancer, accounting for about 85%. As the cost of sequencing continues to decrease and the amount of data continues to grow, GWAS and NGS as the main techniques to find disease-causing genes are time-consuming and laborious, and machine learning methods are getting more and more attention. In the paper, we proposed a new network-based method that is integrated with two different graph embedding methods to identify genes related to NSCLC. In order to learn about the relationships between genes and diseases, we first built a gene interaction network made up of both relevant and unrelated genes to the NSCLC disease. Then, we utilized deep walk and GCN to learn gene–disease interactions. Finally, DNN was constructed as the prediction module. This method concerns the gene network topology relationship and is conducive to mining genetic characteristics. We compared our method with several other methods and demonstrated better performance of our method.

We did case studies on new samples to verify the effectiveness of deepRW. We found that tumor protein p63(TP63) is related to NSCLC. Gürgen et al. (33) found that TP63 expression values were higher than the predefined cutoff of 12 in 23 NSCLC tumors with squamous cell carcinoma histology. general transcription factor IIH subunit 4(GTF2H4) was also found and supported by Wang et al. (34) who reported that GTF2H4 is associated with lung cancer risk.

Compared with machine learning methods, deepRW as a deep learning method needs more time and more samples to train to obtain better performance. In the future, we will study the ability of deepRW to identify other pathogenic genes.

## Data availability statement

The datasets presented in this study can be found in online repositories. The names of the repository/repositories and accession number(s) can be found in the article/**Supplementary Material**.

## Ethics statement

Ethical review and approval was not required for the study on human participants in accordance with the local legislation and institutional requirements. Written informed consent for participation was not required for this study in accordance with the national legislation and the institutional requirements.

## Author contributions

YCa, YL, and JW conceived and designed study, collected and analyzed data. QW and YCh statistical analyses. YCa, YL, and YCh drafted and edited manuscript. All authors contributed to the article and approved the submitted version.

## Conflict of interest

The authors declare that the research was conducted in the absence of any commercial or financial relationships that could be construed as a potential conflict of interest.

## Publisher’s note

All claims expressed in this article are solely those of the authors and do not necessarily represent those of their affiliated organizations, or those of the publisher, the editors and the reviewers. Any product that may be evaluated in this article, or claim that may be made by its manufacturer, is not guaranteed or endorsed by the publisher.

## References

[1] Zitnik M Agrawal M Leskovec J . Modeling polypharmacy side effects with graph convolutional networks. Bioinformatics (2018) 34:i457–66. doi: 10.1093/bioinformatics/bty294 PMC6022705 29949996

[2] Navada S Lai P Schwartz A Kalemkerian G . Temporal trends in small cell lung cancer: Analysis of the national surveillance, epidemiology, and end-results (SEER) database. J Clin Oncol (2006) 24:7082–2. doi: 10.1200/jco.2006.24.18_suppl.7082

[3] Matakidou A Eisen T Houlston R . Systematic review of the relationship between family history and lung cancer risk. Br J Cancer (2005) 93:825–33. doi: 10.1038/sj.bjc.6602769 PMC2361640 16160696

[4] Hung RJ Mckay JD Gaborieau V Boffetta P Hashibe M Zaridze D . A susceptibility locus for lung cancer maps to nicotinic acetylcholine receptor subunit genes on 15q25. Nature (2008) 452:633–7. doi: 10.1038/nature06885 18385738

[5] Hu Z Wu C Shi Y Guo H Zhao X Yin Z . A genome-wide association study identifies two new lung cancer susceptibility loci at 13q12. 12 and 22q12. 2 in han Chinese. Nat Genet (2011) 43:792–6. doi: 10.1038/ng.875 21725308

[6] Wang Y Broderick P Webb E Wu X Vijayakrishnan J Matakidou A . Common 5p15. 33 and 6p21. 33 variants influence lung cancer risk. Nat Genet (2008) 40:1407–9. doi: 10.1038/ng.273 PMC2695928 18978787

[7] Sun Y Duan J Fang W Wang Z Du X Wang X . Identification and validation of tissue or ctDNA PTPRD phosphatase domain deleterious mutations as prognostic and predictive biomarkers for immune checkpoint inhibitors in non-squamous NSCLC. BMC Med (2021) 19:1–19. doi: 10.1186/s12916-021-02075-5 34615542 PMC8496052

[8] Liu Y Kheradmand F Davis CF Scheurer ME Wheeler D Tsavachidis S . Focused analysis of exome sequencing data for rare germline mutations in familial and sporadic lung cancer. J Thorac Oncol (2016) 11:52–61. doi: 10.1016/j.jtho.2015.09.015 26762739 PMC4714038

[9] Rao A Vg S Joseph T Kotte S Sivadasan N Srinivasan R . Phenotype-driven gene prioritization for rare diseases using graph convolution on heterogeneous networks. BMC Med Genomics (2018) 11:1–12. doi: 10.1186/s12920-018-0372-8 29980210 PMC6035401

[10] Han P Yang P Zhao P Shang S Liu Y Zhou J . (2019). GCN-MF: disease-gene association identification by graph convolutional networks and matrix factorization, in: Proceedings of the 25th ACM SIGKDD international conference on knowledge discovery & data mining), pp. 705–13. Available at: https://dblp.org/rec/conf/kdd/HanYZSLZ0K19.html

[11] Wang X Gong Y Yi J Zhang W . (2019). Predicting gene-disease associations from the heterogeneous network using graph embedding, in: 2019 IEEE international conference on bioinformatics and biomedicine (BIBM): IEEE), . pp. 504–11. Available at: http://ieeebibm.org/BIBM2019/AcceptedPapers.html

[12] Zhao T Liu J Zeng X Wang W Li S Zang T . Prediction and collection of protein–metabolite interactions. Briefings Bioinf (2021) 22:bbab014. doi: 10.1093/bib/bbab014 33554247

[13] Cheng N Chen C Li C Huang J . Inferring cell-type-specific genes of lung cancer based on deep learning. Curr Gene Ther (2022), 1–6. doi: 10.2174/1566523222666220324110914 35331109

[14] Li Y Kuwahara H Yang P Song L Gao XJB . PGCN: Disease gene prioritization by disease and gene embedding through graph convolutional neural networks. bioRxiv (2019) 532226. doi: 10.1101/532226

[15] Kipf TN Welling M . (2016)., Semi-supervised classification with graph convolutional networks, Published as a conference paper at ICLR 2016. Available at: https://arxiv.org/abs/1609.02907

[16] Zhao T Hu Y Cheng L . Deep-DRM: a computational method for identifying disease-related metabolites based on graph deep learning approaches. Brief Bioinform (2020) bbaa212. doi: 10.1093/bib/bbaa212 33048110

[17] Xiong Y Guo M Ruan L Kong X Tang C Zhu Y . Heterogeneous network embedding enabling accurate disease association predictions. BMC Med Genomics (2019) 12:1–17. doi: 10.1186/s12920-019-0623-3 31865913 PMC6927100

[18] Yu L Shen X Zhong D Yang J . Three-layer heterogeneous network combined with unbalanced random walk for miRNA-disease association prediction. Front Genet (2019) 10:1316. doi: 10.3389/fgene.2019.01316 31998371 PMC6967737

[19] Zhao T Lyu S Lu G Juan L Zeng X Wei Z . SC2disease: a manually curated database of single-cell transcriptome for human diseases. Nucleic Acids Res (2021) 49:D1413–9. doi: 10.1093/nar/gkaa838 PMC7778914 33010177

[20] Zeng X Zhu S Liu X Zhou Y Nussinov R Cheng F . deepDR: A network-based deep learning approach to *in silico* drug repositioning. Bioinformatics (2019) 35:5191–8. doi: 10.1093/bioinformatics/btz418 PMC6954645 31116390

[21] Perozzi B Al-Rfou R Skiena S . (2014). Deepwalk: Online learning of social representations, in: Proceedings of the 20th ACM SIGKDD international conference on Knowledge discovery and data mining) pp. 701–10. doi: 10.1145/2623330.2623732

[22] Zhu L Hong Z Zheng H . (2019). Predicting gene-disease associations *via* graph embedding and graph convolutional networks, in: 2019 IEEE International Conference on Bioinformatics and Biomedicine (BIBM): IEEE), pp. 382–9. Available at: http://ieeebibm.org/BIBM2019/AcceptedPapers.html

[23] Mikolov T Chen K Corrado G Dean J . Efficient estimation of word representations in vector space. (2013). arXiv:1301.3781v3.

[24] Xu B Wang N Chen T Li M . Empirical evaluation of rectified activations in convolutional network. (2015). https://arxiv.org/abs/1505.00853.

[25] Ioffe S Szegedy C . Batch normalization: Accelerating deep network training by reducing internal covariate shift. OALib Journal (2015) 3: 448–456.

[26] Piñero J Bravo À. Queralt-Rosinach N Gutiérrez-Sacristán A Deu-Pons J Centeno E . DisGeNET: A comprehensive platform integrating information on human disease-associated genes and variants. Nucleic Acids Res (2016), 45(D1):D833–D839. 27924018 10.1093/nar/gkw943 PMC5210640

[27] Hwang S Kim CY Yang S Kim E Hart T Marcotte EM . HumanNet v2: human gene networks for disease research. Nucleic Acids Res (2019) 47:D573–80. doi: 10.1093/nar/gky1126 PMC6323914 30418591

[28] Wu C Orozco C Boyer J Leglise M Goodale J Batalov S . BioGPS: an extensible and customizable portal for querying and organizing gene annotation resources. Genome Biol (2009) 10:1–8. doi: 10.1186/gb-2009-10-11-r130 PMC3091323 19919682

[29] Tong H Faloutsos C Pan J-Y . (2006). Fast random walk with restart and its applications, in: Sixth international conference on data mining (ICDM'06): IEEE), pp. 613–22. Available at: https://ieeexplore.ieee.org/document/4053087

[30] Gönen M Kaski S . Kernelized Bayesian Matrix Factorization. IEEE Trans Pattern Anal Mach Intell (2014) 36(10):2047–60. doi: 10.1109/TPAMI.2014.2313125 26352634

[31] Breiman L . Random forests. Mach Learn (2001) 45:5–32. doi: 10.1023/A:1010933404324

[32] Li G Muller M Thabet A Ghanem B . (2019). Deepgcns: Can gcns go as deep as cnns?, in: Proceedings of the IEEE/CVF international conference on computer vision) pp. 9267–76. Available at: https://dblp.uni-trier.de/rec/conf/iccv/Li0TG19.html

[33] Gürgen D Conrad T Becker M Sebens S Röcken C Hoffmann J . breaking the crosstalk of the cellular tumorigenic network by low-dose combination therapy in lung cancer patient-derived xenografts. Commun Biol (2022) 5:1–10. 35039644 10.1038/s42003-022-03016-5 PMC8763947

[34] Wang M Liu H Liu Z Yi X Heike B Hung RJ . Genetic variant in DNA repair gene GTF2H4 is associated with lung cancer risk: A large-scale analysis of six published GWAS datasets in the TRICL consortium. Carcinogenesis (2016), 37(9):888–896. 27288692 10.1093/carcin/bgw070 PMC5008248

